# In Vitro Antiviral Effects of Green-Lipped Mussel Oil and Low-Molecular-Weight Fucoidan on HSV, RSV, and SARS-CoV-2 Pseudovirus

**DOI:** 10.3390/biomedicines14061184

**Published:** 2026-05-23

**Authors:** Belgheis Ebrahimi, Xu Cindy Yang, Carol Wang, Yiming Yue, Johnson Liu, Jun Lu, John A. Taylor

**Affiliations:** 1 Auckland Bioengineering Institute, University of Auckland, Auckland 1142, New Zealand; bebr067@aucklanduni.ac.nz (B.E.);; 2 School of Biological Sciences, University of Auckland, Auckland 1142, New Zealand; 3 School of Biomedical Sciences, Faculty of Medicine and Health, University of New South Wales, Sydney 2052, Australia; johnson.liu@unsw.edu.au; 4 School of Chemical Sciences, University of Auckland, Auckland 1142, New Zealand; 5 Future Food and Agriculture Department, Yangtze Delta Region Institute of Tsinghua University, Jiaxing 314006, China

**Keywords:** fucoidan, mussel, extract, virus, *Undaria pinnatifida*, *Perna canaliculus*

## Abstract

**Background/Objectives:** Marine-derived bioactive compounds have attracted increasing interest due to their potential antiviral properties. This study investigated in vitro antiviral activity of oil extracted from the green-lipped mussel (*Perna canaliculus*, GLM) and low-molecular-weight (LMW) fucoidan from *Undaria pinnatifida* against three human viruses in mammalian cell systems. herpes simplex virus-1 (HSV-1), respiratory syncytial virus (RSV), and SARS-CoV-2. These marine compounds were selected with the longer-term aim of evaluating their combination as a potential synergistic antiviral strategy. **Methods:** Antiviral efficacy was assessed using complementary assay platforms, including plaque reduction assays in mammalian cell systems and a lentiviral pseudovirus system delivering a bioluminescent reporter gene in HEK293/ACE2 cells pseudotyped with the SARS-CoV-2 spike glycoprotein. Cytotoxicity was assessed in parallel, and the selectivity index (SI) was calculated as the ratio of CC_50_ to IC_50_ for each compound and virus tested. **Results:** GLM oil showed potential antiviral activity against SARS-CoV-2 pseudovirus (SI > 6.20), with limited activity against RSV (SI > 3.48) and HSV-1 (SI > 2.28). In contrast, LMW fucoidan did not demonstrate antiviral activity against any of the tested viruses. **Conclusions:** These findings support further investigation of GLM-derived bioactive compounds as potential antiviral agents, including studies to elucidate their mechanisms of action and in vivo studies to confirm their antiviral efficacy. Combination studies were not pursued in the present work as both compounds require further optimisation individually; however, future studies should evaluate their combined antiviral potential, as synergistic or additive effects remain plausible.

## 1. Introduction

Viral infections remain a major cause of mortality and morbidity in both human and animal populations worldwide. Respiratory viral infections, in particular, continue to pose significant global health challenges, as highlighted by the recent COVID-19 pandemic caused by the novel betacoronavirus, SARS-CoV-2 [1]. In addition to vaccine development, there is an urgent need to identify effective antiviral agents to aid in the prevention and management of COVID-19. Another important respiratory pathogen, respiratory syncytial virus (RSV), is a leading cause of pediatric hospitalization and acute lower respiratory tract infections in children under two years of age globally [2,3]. Although the nucleoside analogue ribavirin is currently used in antiviral therapy against RSV, its clinical efficacy, overall benefit, and safety remain uncertain [4]. Beyond respiratory viruses, infections targeting non-respiratory tissues continue to present substantial health concerns. Among these, herpes simplex virus (HSV) is a highly prevalent human pathogen responsible for both primary and recurrent infections, which range from mild mucocutaneous lesions to severe, life-threatening disease in immunocompromised individuals. HSV-1 and HSV-2 are the causative agents of orofacial and genital herpes, respectively, and circulate globally throughput the year. Infection is characterized by an initial acute phase followed by the establishment of a life-long latency. Standard treatment replies on nucleoside analogies such as acyclovir, which inhibits viral DNA polymerase. While these therapies are generally effective, prolonged use has contributed to the emergence of drug-resistant viral strains, which account for approximately 5% of HSV infections in immunocompromised patients [5,6]. The persistence of established viral infections, together with the ongoing threat of emerging and re-emerging zoonotic viruses, underscores the critical need to identify new antiviral agents. Although vaccines and direct-acting antiviral drugs remain the principal strategies for prophylactic and therapeutic intervention during sporadic outbreaks and epidemics or pandemics, bioactive compounds and extracts from naturally occurring plants and animals have also demonstrated antiviral activity compatible and may contribute to reducing the burden of viral disease in humans [4,7].

Marine organisms represent a rich source of naturally occurring bioactive compounds with antiviral potential. The exploration and utilisation of marine resources have contributed substantially to their application in modern food and pharmaceutical research and development, owing to their high availability, low toxicity, and diverse health-promoting properties [3,8]. For example, fucoidan, a sulfated polysaccharide derived from brown algae such as *Undaria pinnatifida*, has demonstrated promising antiviral activity, highlighting its potential as a functional food or nutraceutical for the prevention and treatment of viral infections. More broadly, sulfated polysaccharides from marine algae exhibit antiviral effects against a range of viruses, including HSV-1, HSV-2, HCMV, and influenza A [5,9,10,11,12]. These compounds interfere with multiple stages of the viral life cycle, particularly the initial phase of infection—viral entry—which is a critical target for antiviral intervention [13]. In particular, high-molecular-weight fucoidans extracted from *U. pinnatifida* have exhibited inhibitory activity against a range of human viruses in vitro [5,9,10,11,14]. These studies indicate that the antiviral efficacy of fucoidan is influenced by its molecular weight and degree of sulfation [13]. Molecular weight is a critical factor influencing the biological activity of fucoidan. Fucoidans are generally classified into low-, medium-, and high-molecular-weight fractions (LMW, MMW, and HMW) [15]. Among these, MMW and HMW fucoidans often exhibit poor absorption and limited bioavailability due to their high viscosity and low permeability, which restricts their practical applications [16]. In contrast, LMW fucoidan is more readily absorbed and demonstrates greater potential for use in both pharmaceutical and functional food applications [15]. Previous studies support the enhanced bioactivity of LMW fucoidan. For example, Sun et al. (2018) reported that LMW fucoidan derived from *Laminaria japonica* significantly prolonged the survival time of mice infected with Human Parainfluenza Virus type 1 [17]. However, to date, no studies have specifically examined the antiviral potential of LMW fucoidan derived from *U. pinnatifida*. Nevertheless, existing evidence indicates that LMW fucoidan generally exhibits stronger bioactivity than its HMW fractions or crude fucoidan [18,19,20]. Based on this evidence, the present study investigates the antiviral activity of LMW fucoidan derived from *U. pinnatifida*.

Another marine organism recognised for its health-promoting bioactive compounds is the green-lipped mussel (GLM), *Perna canaliculus*. GLM-derived products have been widely investigated as alternative or complementary treatments for inflammatory conditions, including asthma, osteoarthritis, and rheumatoid arthritis [21]. In particular, GLM extracts, most notably the extracted oil, have demonstrated anti-arthritic and anti-inflammatory effects in both in vitro and in vivo models [22,23,24,25,26].

Respiratory viral infections such as COVID-19 and RSV are associated with significant inflammatory responses that contribute to cardiovascular complications. Excessive immune activation can lead to the overproduction of proinflammatory cytokines, resulting in a cytokine storm and subsequent multi-organ damage [27]. Bioactive lipids, particularly PUFAs, play a crucial role in the resolution of inflammation and in regulating both acute and chronic inflammatory processes [28]. Omega-3 fatty acid intake has been associated with reduced levels of circulating inflammatory markers in patients with severe COVID-19 [29]. In addition to their immunomodulatory effects, emerging evidence suggests that PUFAs may exert direct antiviral activity by interfering with multiple stages of the viral life cycle, including viral entry, intracellular localization, and replication [30,31,32]. For instance, EPA and DHA have been shown to inhibit hepatitis C virus replication [30]. Similarly, free fatty acids such as arachidonic acid, oleic acid, and linoleic acid exhibit antiviral activity against several enveloped viruses, including influenza, HSV, Sindbis, and Sendai virus [33,34]. Furthermore, alpha-linolenic acid (ALA) has demonstrated dose-dependent inhibition of Zika virus replication by disrupting early stages of the viral cycle, such as viral binding, adsorption, and entry, likely through interactions with the viral membrane [35].

GLM oil extract is rich in polyunsaturated fatty acids (PUFAs), compounds known to possess anti-inflammatory properties and, in some cases, the ability to interfere with viral entry into host cells [36,37]. Based on these properties, we hypothesised that GLM oil may also exhibit antiviral activity. Using a range of in vitro infection assays designed to measure viral entry into mammalian cells, we present novel data on the antiviral potential of two marine-derived bioactive extracts.

In this study, we hypothesized that LMW fucoidan and GLM oil, either individually or in combination, possess antiviral activities. We aimed to evaluate the antiviral effects of GLM oil derived from *P. canaliculus* and LMW fucoidan from *U. pinnatifida*, against HSV-1, a SARS-CoV-2 pseudovirus, and RSV, using Vero, HEK293/ACE2, and HEp-2 cell lines, respectively. The primary objective was to screen the individual antiviral potential of these marine-derived compounds and assess their suitability as natural antiviral agents. The findings presented here provide a foundation for future studies examining their combined use, as previous research suggests that combinations of marine bioactive compounds may confer synergistic effects and broader therapeutic benefits [38,39].

## 2. Materials and Methods

### 2.1. Chemical Composition of GLM Oil and Fucoidan

GLM oil used in this study was extracted from commercially available *P. canaliculus* powder obtained from the Nelson Greenshell Mussel Farm) using an organic solvent extraction method comprising methyl tert-butyl ether and methanol. The detailed chemical composition of this extract has been reported previously [23]. Lipid class analysis revealed that the extract consisted of aliphatic hydrocarbons (3.7%), triacylglycerols (25.39%), free fatty acids (35.84%), sterols (7.50%), and phospholipids (27.56%). Gas chromatography–mass spectrometry (GC-MS) identified a range of saturated, monounsaturated, and PUFAs. The most abundant FFAs included eicosapentaenoic acid (EPA; 20:5n-3), palmitic acid (16:0), docosahexaenoic acid (DHA; 22:6n-3), and palmitoleic acid (16:1n-7). PUFAs constituted the largest lipid fraction (64%), with omega-3 fatty acids accounting for 38.29% of the total lipid content [23,40]. For cell-based assays, GLM oil was initially diluted in absolute ethanol to yield a 2% (*v*/*v*) stock solution and subsequently diluted in cell culture medium. The final ethanol concentration (2%) was confirmed to have no effect on cell viability, as previously reported [40].

LMW fucoidan used in this study was derived from *U. pinnatifida* harvested in New Zealand and had a molecular weight ranging from 1 to 10 kDa. Its composition included 2.76 ± 0.31% total sugars, 1.42 ± 0.03% uronic acids, 7.04 ± 0.47% amino acids, and 16.62 ± 1.31% sulfate. A commercial fucoidan sample derived from *U. pinnatifida* (≥95%, CAS: 9072-19-9) was purchased from Sigma-Aldrich. It contains 96.83 ± 1.71% total sugar, 1.61 ± 0.06% uronic acid, and 25.59 ± 0.63% sulfate. The chemical characteristics and biological activities of this LMW fucoidan preparation and commercial fucoidan have been previously described [41,42].

### 2.2. Cells and Viruses

HEp-2 and Vero (CCL-81) were obtained from ATCC. BSR-T7/5 cells were a gift from Karl-Klaus Conzelmann (Max von Pettenkofer-Institute of Virology, Ludwig-Maximilians-Universität München). Cells were maintained in Dulbecco’s Modified Eagle’s Medium (DMEM) containing 4 mM L-glutamine, 4500 mg/L glucose, 1 mM sodium pyruvate, and 1500 mg/L sodium bicarbonate, supplemented with 10% fetal bovine serum (FBS; Moregate Biotech, Auckland, New Zealand), 50 I.U./mL penicillin and 100 μg/mL streptomycin. Lenti-X^TM^ 293T (TaKaRa Bio, San Jose, CA, USA) is a subclone of the transformed human embryonic kidney cell line. ACE2-HEK293 cells were generated by stable transfection of wild-type HEK-293 cells with the plasmid pSB-bi-Puro, which utilises the Sleeping Beauty transposase system to insert cDNA encoding human ACE2 into the host genome [43]. Lenti-X^TM^ 293T and ACE2-HEK293 cells were cultured in DMEM containing 10% heat-inactivated fetal bovine serum (56 °C for 30 min to inactivate complement activity). ACE2-HEK293 cells were maintained in media containing 2 μg/mL puromycin.

The KOS strain of HSV-1 was obtained from Professor Tony Cunningham (Westmead Institute, University of Sydney), propagated in Vero cells and titrated by plaque assay as previously described [44]. A recombinant version of RSV expressing a fluorescent reporter was generated by transfection of BSRT7/5 cells with a bacterial artificial chromosome (BAC) containing the antigenomic cDNA based on a chimeric RSV genotype A2-line19F, together with four helper plasmids encoding N, P, L and M2-1 proteins, optimized for human codon bias. All plasmids were obtained from BEI Resources (NIAID). The BAC construct (pSynkRSV-I19F) contains the gene encoding the red fluorescent protein monomeric Katushka 2 (mKate2) in the first gene position, flanked by RSV regulatory elements [45]. Recombinant fluorescent RSV was rescued in BSR-T7/5 cells to generate a P0 stock, which was amplified by serial passage in HEp-2 cells. All experiments were performed with P3 virus stocks.

### 2.3. Cell Viability Assay

Cell viability of ACE2-HEK293, Vero, and HEp-2 cells following exposure to each extract was assessed using the MTT assay. Briefly, 5 × 10^4^ cells were seeded into 96-well plates and incubated overnight. The following day, cells were treated with varying concentrations of extracts (5–400 µg/mL) and incubated for the same duration as used in the antiviral assays. After treatment, the medium was aspirated and replaced with 100 µL of fresh DMEM, followed by additional 24 h incubation. Four hours prior to endpoint measurement, MTT solution (5 mg/mL) was added to each well. After removal of the supernatant, the resulting formazan crystals were dissolved in dimethyl sulfoxide (DMSO), and absorbance was measured at 540 nm using a microplate reader (SPARK 10M- Tecan Trading AG, Mannedorf, Switzerland). Cell viability was expressed relative to untreated control cells. All experiments were performed in triplicate [46].

### 2.4. Production of SARS-CoV-2 Spike Pseudotyped Lentivirus

Lentivirus vectors were produced as previously described [47], except that plasmids were complexed with Lipofectamine 3000 instead of PEI. Lenti-X^TM^ 293T cells were seeded in 6-well plates at a density of 8 × 10^5^ cells per well in growth media and incubated overnight at 37 °C. The following day, the medium was replaced with 2.5 mL Opti-MEM (Gibco, Waltham, MA, USA) supplemented with 5% heat-inactivated serum and 1% sodium pyruvate (Gibco), and cells were transfected with plasmid DNA using the LF3000 according to the manufacturer’s instructions. After 72 h, supernatants containing lentivirus particles were harvested, filtered through a 0.2 µm membrane, and stored at −80 °C in 0.2 mL aliquots.

### 2.5. Lentivirus Transduction of ACE2-HEK293 Cells and Luciferase Assay

ACE2-HEK293 cells were detached using Accutase™ cell dissociation reagent (Thermofisher Scientific, Waltham, MA, USA) and seeded into poly-L-lysine-coated 96-well plates at a density of 1.4 × 10^4^ cells per well. The following day, lentivirus stocks were thawed on ice and diluted 1:20 in infection medium (Opti-mem supplemented with 5% heat inactivated FBS; 1% sodium pyruvate). In parallel, test extracts were serially diluted in infection medium to 2X the desired final concentrations (0, 25, 50, 100, 200, and 400 μg/mL), mixed 1:1 with diluted lentivirus, and incubated for 45 min at room temperature. Culture medium was then removed from the cells and replaced with 90 μL of each extract-vector mixture. Cells were incubated at 37 °C for 60 h, after which luciferase activity was measured as relative luciferase units (RLU) using Bright-Glo™ Luciferase Assay System and a SpectraMax luminometer (Promega, Madison, WI, USA). RLU values obtained in the presence of extracts were normalised to the control (no extract).

### 2.6. Calculation of IC_50_, CC_50_, and Selectivity Index

To further evaluate antiviral potential, the selectivity index (SI) was calculated. The SI reflects the balance between antiviral efficacy and cytotoxicity and is defined as the ratio of the 50% cytotoxic concentration (CC_50_), which reduces host cell viability by 50%, to the 50% inhibitory concentration (IC_50_), the concentration required to inhibit 50% of viral replication. A higher SI value indicates that a compound can effectively suppress viral infection at non-toxic concentrations, suggesting greater safety and therapeutic potential [48,49]. For natural products, IC_50_ values below 100 μg/mL are generally considered indicative of promising antiviral activity. In addition, a SI value above 4 is typically regarded as acceptable for natural mixtures [50], while values greater than 10 indicate high selectivity and safety [51]. Conversely, low SI values may suggest limited clinical utility due to cytotoxicity occurring near the effective antiviral dose. Therefore, SI serves as a critical parameter for identifying natural compounds suitable for further antiviral development.

### 2.7. Statistical Analysis

Results are presented as means ± standard deviation (SD). All experiments were performed at least in duplicate and repeated independently two to three times. Statistical analyses were conducted using GraphPad Prism software (Version 10.0.0, Boston, MA, USA). Differences between groups were assessed by one-way analysis of variance (ANOVA) followed by Duncan’s multiple range test. The IC_50_ and CC_50_ values were determined by nonlinear regression analysis (log inhibitor) vs. normalized response, variable slope model).

## 3. Result

### 3.1. GLM Oil Inhibits SARS-CoV-2 Pseudovirus Entry into ACE2-HEK293 Cells

**Figure 1:**
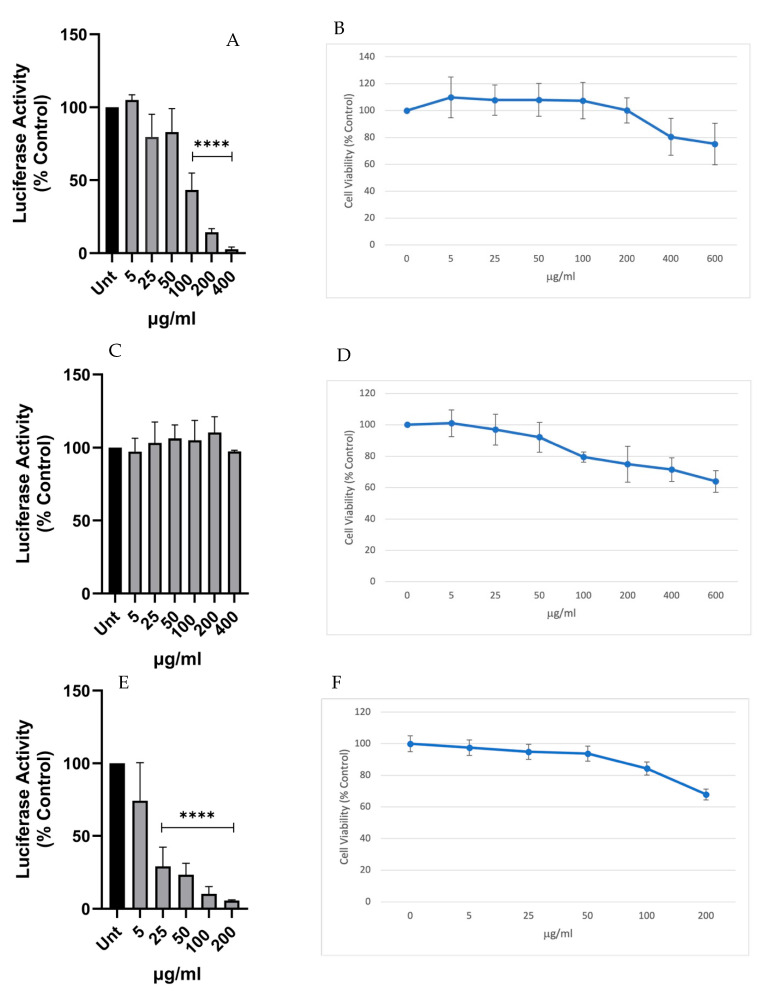
Viral infectivity and cytotoxicity of tested samples against SARS-CoV-2 pseudovirus in ACE2-HEK293 cells. Luciferase activity was used to assess pseudovirus infectivity (**A**,**C**,**E**). ACE2-HEK293 cells were treated with varying concentrations of (**A**) GLM oil, (**C**) LMW fucoidan, and (**E**) commercial fucoidan. Viral infectivity is expressed as a percentage relative to the virus-infected untreated control (Unt: set as 100%). Cell viability was evaluated using the MTT assay (**B**,**D**,**F**). HEK293/ACE2 cells were treated with varying concentrations of (**B**) GLM oil, (**D**) LMW fucoidan, and (**F**) commercial fucoidan. Viability is expressed as a percentage relative to the untreated control (Unt: set as 100%). **** indicates *p* ≤ 0.0001. Data represent the means ± SD from three independent experiments (*n* = 3).

We first evaluated the ability of the extracts to inhibit SARS-CoV-2 pseudovirus infection in ACE2-HEK293 cells. Pseudovirus infection was quantified by measuring luciferase activity 60 h post-infection using the Bright-Glo™ reagent (Figure 1A,C,E). As shown in Figure 1A, GLM oil exhibited dose-dependent activity, reducing luciferase expression or pseudovirus infectivity to 43.33%, 14.33%, and 2.66% at concentrations of 100, 200, and 400 µg/mL, respectively. The half-maximal inhibitory concentration (IC_50_) for GLM oil was calculated as 96.70 µg/mL. In contrast, LMW fucoidan demonstrated negligible inhibition of viral entry at all concentrations tested (Figure 1C). Commercial fucoidan exhibited the most reduction in luciferase activity among all samples (Figure 1E), with an IC_50_ of 11.50 µg/mL. It significantly reduced pseudovirus infectivity to 29.02%, 23.25%, and 10.33% at concentrations of 25, 50, and 100 µg/mL, respectively.

To ensure that the observed reduction in viral infectivity was not attributable to cytotoxic effects, cell viability was assessed using the MTT assay (Figure 1B,D,F). HEK293/ACE2 cells were treated with each sample at concentrations ranging from 5 to 600 µg/mL. After 60 h of treatment, cell viability remained above 80% for all samples at concentrations between 5 and 100 µg/mL. For GLM oil, cell viability decreased slightly with increasing concentration but remained approximately 75% at 600 µg/mL (Figure 1B). In contrast, LMW fucoidan showed a more pronounced reduction in cell viability, reaching 65.33% at 600 µg/mL (Figure 1D). Commercial fucoidan exhibited the highest cytotoxicity, with cell viability decreasing to 68.2% at 200 µg/mL (Figure 1F).

### 3.2. Antiviral Activity Against RSV in HEp-2 Cells

**Figure 2:**
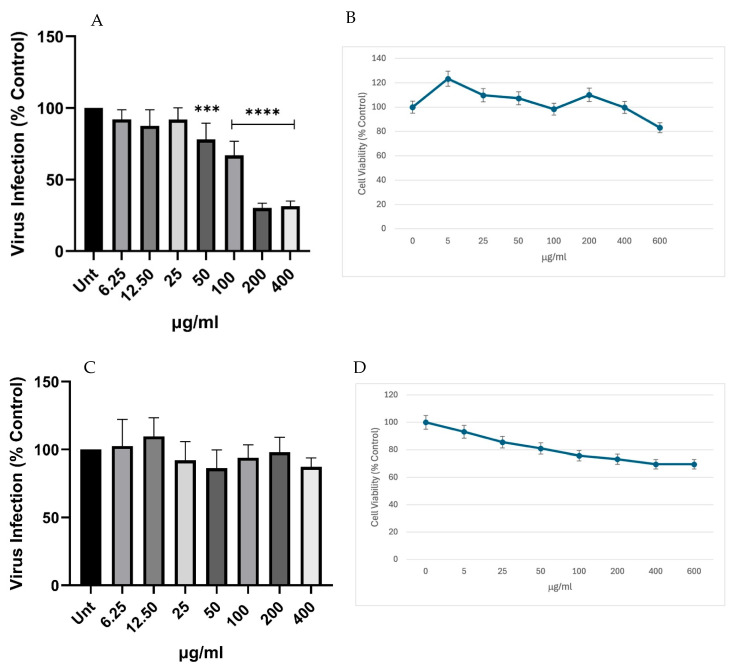
Antiviral activity and cytotoxicity of tested samples against RSV in Hep-2 cells. RSV infectivity was quantified by fluorescence intensity (**A**,**C**). Hep-2 cells were treated with varying concentrations of (**A**) GLM oil and (**C**) LMW fucoidan. Viral infectivity was expressed as a percentage relative to the virus-infected untreated control (Unt: set as 100%). Cell viability was assessed using the MTT assay (**B**,**D**). Hep-2 Cells were treated with varying concentrations of (**B**) GLM oil and (**D**) LMW fucoidan. Viability is expressed as a percentage relative to the untreated control (Unt: set as 100%). *** indicates *p* ≤ 0.001 (**** *p* ≤ 0.0001). Data are presented as mean ± SD from three independent experiments (*n* = 3).

The antiviral activity of GLM oil and LMW fucoidan against RSV infection was evaluated in HEp-2 cells (Figure 2A,C). Infection was quantified by measuring mKate2 fluorescence, which is expressed from the viral genome following viral entry and uncoating [45]. Fluorescence intensity at 24 h post-infection (24 h.p.i) correlated with the viral inoculum, confirming that fluorescence accurately reflected viral infectivity. GLM oil exhibited a clear concentration-dependent reduction in fluorescence at 24 h.p.i. At concentrations of 50, 100, 200, and 400 µg/mL, viral infection was reduced to 78.05%, 66.93%, 30.17%, and 31.40% of the control, respectively (Figure 2A). The IC_50_ of GLM oil was 172.6 μg/mL. In contrast, LMW fucoidan showed only a modest and concentration-independent effect, with the lowest infection level of 86.35% observed at 50 µg/mL, and no significant changes at higher concentrations (Figure 2C).

The MTT assay was performed to assess cell viability and to exclude the possibility that the observed reduction in viral infection was due to sample-induced cytotoxicity (Figure 2B,D). Hep-2 cells were treated with each sample at concentrations ranging from 5 to 600 µg/mL. After 2 h of treatment, GLM oil exhibited minimal cytotoxicity, with cell viability consistently remaining above 80% across all tested concentrations, indicating a favourable safety profile within the effective antiviral range. In contrast, LMW fucoidan caused a more pronounced reduction in cell viability, particularly at higher concentrations, with viability decreasing to 69.49% at 600 µg/mL (Figure 2D). These results suggest that the antiviral activity of GLM oil is unlikely to be attributable to cytotoxic effects, whereas the modest cytotoxicity observed for LMW fucoidan at higher concentrations should be considered when interpreting its limited antiviral efficacy.

### 3.3. Antiviral Activity Against HSV-1 in Vero Cells

**Figure 3:**
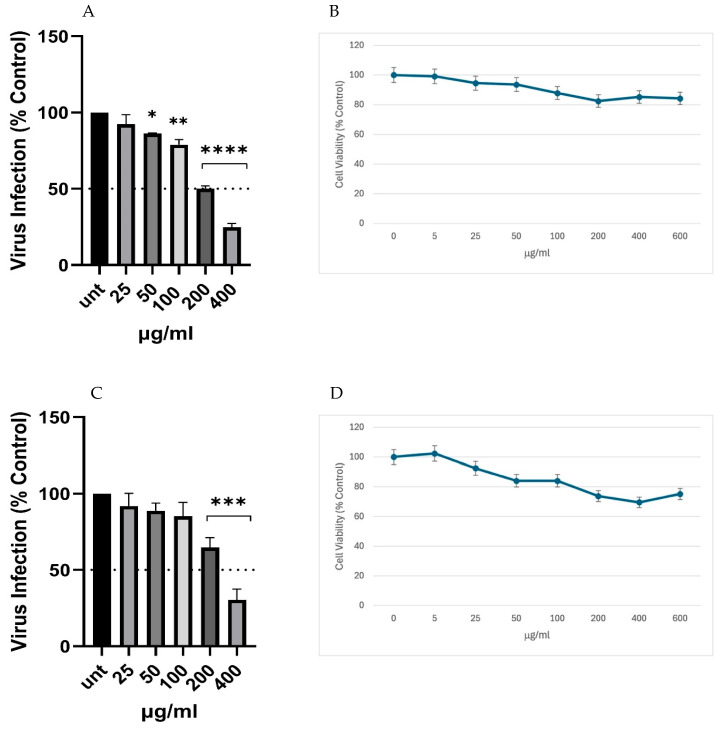
Viral infectivity and cytotoxicity of GLM oil and LMW fucoidan against HSV-1 in Vero cells. HSV infectivity was assessed using a plaque reduction assay (**A**,**C**). Vero cells were treated with varying concentrations of (**A**) GLM oil and (**C**) LMW fucoidan. The viral infectivity was expressed as a percentage relative to the virus-infected untreated control (Unt: set as 100%). Cell viability was evaluated using the MTT assay (**B**,**D**). Vero cells were treated with varying concentrations of (**B**) GLM oil and (**D**) LMW fucoidan. Viability is expressed as a percentage relative to the untreated control (Unt: set as 100%). * indicates *p* ≤ 0.05 (** *p* ≤ 0.01, *** *p* ≤ 0.001, **** *p* ≤ 0.0001). Data are presented as mean ± SD from three independent experiments (*n* = 3).

The antiviral activity against HSV-1 was evaluated in Vero cells using a plaque reduction assay. Results were expressed as a percentage of infection relative to the untreated control to maintain consistency across all assays (Figure 3A,C). GLM oil showed a clear concentration-dependent decrease in infection, with infection levels of 86.27%, 78.92% 50.04%, and 24.78% at 50, 100, 200, and 400 µg/mL, respectively (Figure 3A). This reduction was not attributable to cytotoxicity, as cell viability remained above 80% across all tested concentrations based on MTT assay results (Figure 3B). In comparison, LMW fucoidan also reduced infection, with infection levels of 64.67% at 200 µg/mL and 30.48% at 400 µg/mL (Figure 3C). However, its apparent antiviral effect at higher concentrations may be partially influenced by cytotoxicity. MTT assay results showed that cell viability decreased to 73.7% at 200 µg/mL and 69.51% at 400 µg/mL (Figure 3D), suggesting that the observed reduction in infection may be confounded by reduced cell viability at these concentrations.

### 3.4. Antiviral Activity and Selectivity Index

The SI was calculated as the ratio of CC_50_ to IC_50_. A higher SI indicates lower cytotoxicity and greater antiviral selectivity, suggesting a more favourable safety profile. Cytotoxicity of the GLM oil was assessed using the MTT assay across concentrations ranging from 0 to 600 µg/mL. Cell viability remained above 80% at all tested concentrations in Hep-2 and Vero cells, and above 75% in HEK293/ACE2 cells, with no concentration causing 50% cell death. Accordingly, the CC_50_ was conservatively reported as > 600 µg/mL. Similar results were observed for the LMW fucoidan. SI values were calculated for compounds showing statistically significant inhibition in either the plaque reduction assay or pseudovirus luciferase assay (*p* < 0.05) and are summarised in Table 1. In this study, compounds with an SI value greater than 4 were considered to exhibit acceptable antiviral activity, consistent with previously reported criteria for natural mixtures [50].

## 4. Discussion

### 4.1. Antiviral Activity of Fucoidan and LMW Fraction

#### 4.1.1. Antiviral Activity of LMW Fucoidan Against Respiratory Viruses (SARS-CoV-2 Pseudovirus and RSV)

Fucoidan has attracted considerable interest as a potential antiviral agent for the prevention of COVID-19. The surface of the coronavirus is largely covered by glycosylated spike (S) proteins, which play a crucial role in facilitating viral attachment, mediating membrane fusion, and enabling entry into host cells. Due to its essential role in infection, the spike protein is a key target for vaccine development and antiviral therapies. During the initial stage of infection, the spike protein binds to the host cell’s angiotensin-converting enzyme 2 (ACE2) receptor a membrane-bound glycoprotein highly expressed in tissues such as the lungs, kidneys, heart, and endothelium. This interaction allows the virus to enter the host cell [13,52]. Therefore, effective antiviral agents should aim to block or interfere with this binding process to prevent viral entry. Research suggests that fucoidan can bind to the viral spike glycoprotein, inhibiting its attachment to host cells and thereby exhibiting antiviral activity against SARS-CoV-2 in vitro [13,53,54].

Due to the high risk of SARS-CoV-2 transmission, authentic virus neutralization assays must be conducted in Biosafety level 3 laboratories equipped with negative pressure systems, which limits their accessibility and practicality. As an alternative, pseudotyped viral particles expressing the SARS-CoV-2 spike protein can be safely used in Biosafety level 2 settings. The main focus of this study was to assess whether the tested samples could inhibit the attachment or entry of a SARS-CoV-2 pseudovirus into cells expressing the ACE2 receptor. For this purpose, HEK293 cells engineered to express ACE2 were employed [55] to evaluate the ability of samples to block viral entry by interfering with the interaction between the viral spike glycoprotein and the host ACE2 receptor in vitro. A similar approach was used by Yim et al. (2021), who demonstrated that fucoidan effectively blocked the entry of SARS-CoV-2 pseudovirus into HEK293/ACE2 cells, further supporting its potential to inhibit viral entry [56].

Another viral infection of the respiratory tract is caused by RSV, an enveloped RNA virus that uses its G and F proteins for attachment to and fusion with host cells. Similar to SARS-CoV-2, RSV relies on glycosaminoglycans (GAGs), such as heparan sulfate, as initial attachment factors that facilitate viral entry [57]. Given these shared mechanisms, targeting the early stages of viral entry has emerged as a potential therapeutic strategy. For instance, humanized monoclonal antibodies directed against the RSV G or F proteins have been shown to protect high-risk children from infection [58]. These findings support the strategy of disrupting the interaction between the RSV G-protein and its cellular receptors as an effective means of controlling infection. In line with this, Malhotra et al. [57] reported that fucoidan exhibits potent antiviral activity against RSV, underscoring its potential as a therapeutic agent. Considering the similar reliance of both RSV and SARS-CoV-2 on host cell surface molecules and their shared tropism for respiratory tract, broad-spectrum antiviral agents such as fucoidan, which can inhibit viral attachment through their sulfated structure, may hold promise against both viruses.

In this study, commercial fucoidan extracted from *U. pinnatifida* was used as a control to evaluate the antiviral activity of its LMW fraction against SARS-CoV-2 pseudovirus. As expected, commercial fucoidan significantly reduced pseudovirus infectivity, with an SI greater than 11.37, which is considered indicative of antiviral activity and high selectivity for natural compounds [51]. This effect may be associated with fucoidan’s ability to interfere with the interaction between the viral spike protein and the host cell ACE2 receptor, as suggested in previous studies [53,54,59]. In contrast, the LMW fucoidan fraction showed no effect on pseudovirus infectivity in the same in vitro model. This finding aligns with studies indicating that HMW fucoidan (~100 kDa), particularly those with branched structures, exhibits greater antiviral effects than lower molecular weight fractions (~12 kDa) [53,59]. Similarly, when LMW fucoidan showed no significant reduction in RSV infectivity in Hep-2 cells line, suggesting that this fraction is ineffective at inhibiting RSV infection under conditions tested.

The antiviral activity of fucoidan has been primarily associated with its molecular weight and degree of sulfation [13,60,61]. Higher molecular weight generally provides a greater number of potential binding sites, allowing for more extensive interactions with viral targets. This multipoint binding increases the overall binding strength (avidity), enhancing the compound’s ability to block viral entry into host cells [53]. In contrast, lower molecular weight fucoidan may lack sufficient binding sites or spatial flexibility, resulting in weaker antiviral effects In addition to molecular weight, the degree of sulfation is a critical determinant of antiviral activity of fucoidan [59]. In the present study, the absence of antiviral effect in LMW fucoidan may be attributed to its relatively low sulfation level (~16%). This limitation likely reduces its capacity to inhibit viral infection. The antiviral mechanism of fucoidan and other negatively charged sulfated polysaccharides is largely based on their ability to block viral attachment by binding to positively charged regions on viral envelope glycoproteins, thereby preventing interaction with glycosaminoglycans on the host cell surface [62,63]. Importantly, both the degree of sulfation and the distribution of sulfate groups are key factors influencing antiviral efficacy. Polysaccharides with low sulfation are generally inactive [63], whereas highly sulfated compounds exhibit strong inhibition of viral adsorption [62]. This aligns with findings by Sun et al. [7], who highlighted the importance of high sulfate content for fucoidan’s antiviral activity. Therefore, the limited antiviral effect in our LMW fucoidan sample is most likely due to its low sulfate content, which reduces its ability to interfere with viral entry.

#### 4.1.2. Antiviral Activity of LMW Fucoidan Against HSV

HSV entry into host cells is a multistep process that begins with viral attachment to specific receptors on the cell surface, followed by fusion of the viral envelope with the host cell membrane. In both HSV-1 and HSV-2, initial attachment is mediated by interactions between viral envelope glycoproteins and heparan sulfate proteoglycans (HSPGs) on host cells. This early binding step is critical for successful viral entry and represents a key target for antiviral strategies aimed at blocking infection at its earliest stage [64,65].

The antiviral activity of LMW fucoidan against HSV was evaluated using Vero cell lines. In this study, HSV-1 was pretreated with LMW fucoidan prior to infection, following a protocol similar to that described by Sinha et al. [66]. A significant reduction in plaque formation was observed at concentrations of 200 and 400 µg/mL. These findings are consistent with previous studies demonstrating that fucoidan inhibits HSV infection during the early stages of the viral life cycle. For instance, Sinha et al. [66] and Sun et al. [7] reported that pretreatment of the virus with fucoidan significantly reduced plaque formation, suggesting interference with viral adsorption or penetration into host cells. Similarly, Lee et al. (2004) reported that fucoidan derived from *U. pinnatifida* inhibited HSV by blocking the entry of enveloped viruses through disruption of virus–host cell interactions [7,9,66]. However, the antiviral potency of the LMW fraction was limited, as reflected by the low SI (SI ≥ 2.88) and the reduction in cell viability observed at higher concentrations (400 µg/mL), suggesting that cytotoxicity may partially contribute to the apparent antiviral effect. Therefore, these findings should be interpreted with caution.

### 4.2. Antiviral Activity of GLM Oil

#### 4.2.1. Antiviral Activity of GLM Oil Against Respiratory Viruses (SARS-CoV-2 Pseudovirus and RSV)

To the best of our knowledge, this is the first study to report the in vitro antiviral activity of GLM oil extract, particularly against SARS-CoV-2 pseudovirus, with limited antiviral activity observed against RSV. GLM oil is a rich source of PUFAs, including EPA, DHA, and ALA, which are well recognized for their anti-inflammatory and antiviral properties [30,31,32,35]. In this study, crude GLM oil containing 64% total PUFAs, specifically EPA (45.36 mg/g), DHA (24.84 mg/g), and ALA (8.3 mg/g), was evaluated for its in vitro antiviral activity [23,40]. A virucidal assay was employed, in which the oil was incubated directly with the virus prior to exposure to host cells, enabling assessment of its ability to neutralise viral infectivity [2]. GLM oil demonstrated statistically significant reduction in pseudovirus infectivity at low concentrations against SARS-CoV-2 pseudovirus. Based on the SI values presented in Table 1, GLM oil exhibited a SI of 6.2 and an IC_50_ of 96.7 µg/mL against SARS-CoV-2 pseudovirus. These findings indicate potential antiviral activity, as IC_50_ values below 100 µg/mL are generally considered indicative of effective activity. Moreover, for natural product-based formulations, a SI value above 4 is typically regarded as acceptable, further supporting the potential of GLM oil as a safe and effective antiviral candidate [50].

Although the mechanism of antiviral activity was not investigated in this study, previous reports on PUFAs provide a plausible explanation for the observed inhibitory effects, as these compounds have been shown to interfere with viral entry mechanisms [36,37]. For example, Goc et al. [37] demonstrated that linoleic acid and EPA inhibit SARS-CoV-2 pseudovirus attachment to the ACE2 receptor by interacting with the spike protein’s receptor-binding domain. Similarly, Huang et al. [36] reported that DHA reduced cellular entry of SARS-CoV-2 pseudovirus by modulating ACE2 expression, while Chiang et al. [67] showed that EPA and DHA can block viral entry in human endothelial progenitor cells. Complementing these findings, molecular docking analyses by McGill et al. [29] identified ALA as a top ligand for binding to the SARS-CoV-2 spike protein. They also demonstrated its interaction with RSV fusion proteins, suggesting that ALA may exert broad-spectrum antiviral effects by directly binding to viral fusion or attachment proteins [29]. The presence of these bioactive fatty acids in GLM oil suggests that similar mechanisms may contribute to the antiviral activity observed here. Further studies are therefore warranted to isolate and characterise the active components of GLM oil, clarify their specific roles in viral inhibition, and confirm these effects through detailed mechanistic investigations.

Against RSV in HEp-2 cells, GLM oil significantly reduced viral infection at non-cytotoxic concentrations (*p* < 0.001). However, SI of 3.48 fell slightly below the commonly suggested threshold of SI > 4 for antiviral activity in natural compounds, indicating limited selectivity. Notably, since cytotoxicity was not reached at the highest tested concentration, the true CC_50_ could not be determined, and the calculated SI therefore represents a minimum estimate. This suggests that the limited selectivity reflects moderate antiviral potency rather than cytotoxic effects.

#### 4.2.2. Antiviral Activity of GLM Oil Against HSV

In the present study, we investigated the antiviral activity of crude GLM oil against HSV-1 using a plaque reduction assay in Vero cells. Pre-incubation of GLM oil with HSV-1 prior to cell infection resulted in a statistically significant (*p* < 0.01), dose-dependent reduction in plaque formation across all tested concentrations (5–400 µg/mL), suggesting direct inhibition of viral infectivity. As previously noted for RSV, the absence of a definable CC_50_ means the SI value (~2.28) represents a minimum estimate, indicating that the limited selectivity is more likely attributable to moderate antiviral potency than to cytotoxic effects.

Despite the limited antiviral selectivity observed against HSV-1, the inhibitory effects of GLM oil may be attributed to its content of PUFAs, particularly ALA, a key component of GLM oil [40]. In support of this, Feng et al. [35] reported that pre-incubation of HSV-1 with ALA prior to infection of Vero cells significantly reduced intracellular expression of viral envelope glycoprotein D, indicating suppression of viral replication. Similar inhibitory effects were observed across multiple enveloped viruses, highlighting the broad-spectrum antiviral potential of ALA. Together, these findings support the hypothesis that PUFAs in GLM oil may interfere with viral attachment, fusion, or replication [35]. Mechanistically, PUFAs may exert antiviral effects through direct disruption of the viral envelope and modulation of membrane dynamics. As lipophilic molecules, fatty acids such as ALA can destabilise the lipid bilayer of enveloped viruses and alter host cell membrane properties, thereby impairing viral entry [68,69]. This is consistent with earlier work by Kohn et al. [34], who demonstrated that unsaturated free fatty acids can inactivate enveloped viruses, including HSV, via disruption of the viral envelope. Overall, the limited antiviral selectivity observed against HSV-1 may reflect the complex composition of GLM oil, where active components such as PUFAs may be diluted or antagonised by other constituents. Further fractionation and purification of GLM oil would therefore be necessary to isolate and characterise the active antiviral components.

### 4.3. Limitations and Future Directions

This study provides valuable insights into the biological potential of LMW fucoidan and GLM oil; however, several areas require further investigation to fully establish their therapeutic relevance. A key limitation is the inability to accurately determine the SI, due to the absence of a clearly defined CC_50_ value, as no cytotoxicity was observed at the highest concentrations tested. Consequently, the reported SI represents a conservative minimum estimate and may underestimate the true antiviral potential of the compounds. This limitation restricts a comprehensive evaluation of the therapeutic window and safety profile., Future studies should therefore assess a broader concentration range to enable precise determination of both CC_50_ and IC_50_ values, which is essential for accurately evaluating efficacy and cytotoxicity. In addition, the molecular mechanisms underlying the observed antiviral effects were not investigated. Future research should focus on elucidating these mechanisms using approaches such as time-of-addition assays [7], in which compounds are administered at different stages of the viral life cycle (pre-infection, during viral entry, and post-infection) to identify the most sensitive phase and clarify the mode of action.

The structural characteristics of fucoidan also play a crucial role in its antiviral activity. The LMW fucoidan used in this study exhibited relatively low sulfate content, a key determinant of bioactivity. Accordingly, future work should prioritise optimised extraction and purification methods that preserve or enhance sulfate groups to maximise antiviral efficacy. Similarly, as the GLM oil examined here was in crude form, future studies should isolate and characterise the specific lipid classes responsible for its antiviral activity. Identifying these active components will improve mechanistic understanding, enhance batch consistency, and support the development of more potent formulations through enrichment of bioactive lipids. Finally, given that LMW fucoidan did not demonstrate antiviral activity, combination studies were not pursued in the present work. Our findings suggest that both compounds may require further optimisation, as previously discussed, before a combination approach can be meaningfully evaluated. Nevertheless, their combination warrants future investigation, as these compounds likely act through distinct mechanisms, making synergistic or additive antiviral effects plausible. This is consistent with our previous findings, which demonstrated that combinations of marine-derived bioactives, including LMW fucoidan and GLM oil, can enhance overall efficacy and broaden their therapeutic potential [38,39,70]. Future studies should extend these findings to relevant in vivo models of viral infection to evaluate the antiviral efficacy of GLM oil and LMW fucoidan. For SARS-CoV-2, hACE2 transgenic mice infected with SARS-CoV-2 have been shown to closely mimic key features of human COVID-19 [71]. For HSV-1, murine infection models are commonly used for initial antiviral efficacy assessment, while guinea pig models, which better reflect the latency and recurrence of human disease, are considered the gold standard for therapeutic evaluation [72]. For RSV, established BALB/c mouse models using intranasal viral inoculation can be used to evaluate in vivo antiviral efficacy [73]. These in vivo studies would be essential before advancing to clinical trials to confirm antiviral efficacy and establish the safety profile of these compounds.

## 5. Conclusions

GLM oil demonstrated antiviral activity against SARS-CoV-2 pseudovirus and limited antiviral activity against HSV-1 and RSV. Further investigation is required to identify the specific bioactive components within the crude oil and to elucidate their mechanisms of action. In vivo validation using established animal models of SARS-CoV-2, HSV-1, and RSV infection, followed by early-phase clinical studies, will be essential to confirm antiviral efficacy, safety, and translational potential in humans. LMW fucoidan was evaluated as a potential partner compound for a proposed combination strategy with GLM oil. However, it did not exhibit antiviral activity against the tested viruses, whereas commercial fucoidan, used as a reference, demonstrated antiviral activity against SARS-CoV-2. This difference may be related to structural and compositional features known to influence fucoidan bioactivity. Nevertheless, the specific contribution of these factors was not examined in the present study. Future studies should therefore compare fucoidan fractions with varying molecular weights and sulfate contents to better define the structural characteristics associated with antiviral activity. Finally, combination studies were not pursued in the present work as both compounds require further optimisation individually. Future studies should therefore explore optimised fractions of GLM oil and LMW fucoidan before evaluating their combined antiviral potential, as synergistic or additive effects remain plausible given their distinct mechanisms of action.

## Figures and Tables

**Table 1:** IC_50_ and CC_50_ values and selectivity index (SI).

Compounds	Virus/Cells	CC_50_	IC_50_	SI
Commercial Fucoidan	SARS-CoV-2 pseudovirus/ HEK293-ACE2	>200	11.5	>17.39
GLM oil	SARS-CoV-2 pseudovirus/ HEK293-ACE2	>600	96.7	>6.20
	RSV/Hep-2	>600	172.6	>3.48
	HSV/Vero Cells	>600	263.4	>2.28
LMW Fucoidan	HSV/Vero Cells	>600	208	>2.88

CC_50_: concentration of the compound required to reduce cell viability by 50%; IC_50_: concentration of the compound required to reduce viral infection by 50%. Both CC_50_ and IC_50_ are expressed in μg/mL. The SI for each compound was calculated as the ratio of CC_50_ to IC_50_.

## Data Availability

Data will be made available via request to the corresponding authors.

## References

[1] Zhu N., Zhang D., Wang W., Li X., Yang B., Song J., Zhao X., Huang B., Shi W., Lu R. (2020). A Novel Coronavirus from Patients with Pneumonia in China, 2019. N. Engl. J. Med..

[2] Jana S., Dyna A.L., Pal S., Mukherjee S., Bissochi I.M.T., Yamada-Ogatta S.F., Darido M.L.G., Oliveira D.B.L., Durigon E.L., Ray B. (2024). Anti-respiratory syncytial virus and anti-herpes simplex virus activity of chemically engineered sulfated fucans from Cystoseira indica. Carbohydr. Polym..

[3] Zhang J., Wang S., Yang M., Ding J., Huang Y., Zhu Y., Zhou M., Yan B. (2024). Antiviral activity of a polysaccharide from Sargassum fusiforme against respiratory syncytial virus. Int. J. Biol. Macromol..

[4] Wang H., Ooi E.V., Ang P.O. (2008). Antiviral activities of extracts from Hong Kong seaweeds. J. Zhejiang Univ. Sci. B.

[5] Cooper R., Dragar C., Elliot K., Fitton J.H., Godwin J., Thompson K. (2002). GFS, a preparation of Tasmanian Undaria pinnatifida is associated with healing and inhibition of reactivation of Herpes. BMC Complement. Altern. Med..

[6] Field H.J. (2001). Herpes simplex virus antiviral drug resistance--current trends and future prospects. J. Clin. Virol..

[7] Sun Q.L., Li Y., Ni L.Q., Li Y.X., Cui Y.S., Jiang S.L., Xie E.Y., Du J., Deng F., Dong C.X. (2020). Structural characterization and antiviral activity of two fucoidans from the brown algae Sargassum henslowianum. Carbohydr. Polym..

[8] Molinski T.F., Dalisay D.S., Lievens S.L., Saludes J.P. (2009). Drug development from marine natural products. Nat. Rev. Drug Discov..

[9] Lee J.B., Hayashi K., Hashimoto M., Nakano T., Hayashi T. (2004). Novel antiviral fucoidan from sporophyll of Undaria pinnatifida (Mekabu). Chem. Pharm. Bull..

[10] Hayashi K., Nakano T., Hashimoto M., Kanekiyo K., Hayashi T. (2008). Defensive effects of a fucoidan from brown alga Undaria pinnatifida against herpes simplex virus infection. Int. Immunopharmacol..

[11] Thompson K.D., Dragar C. (2004). Antiviral activity of Undaria pinnatifida against herpes simplex virus. Phytother. Res..

[12] Wozniak M., Bell T., Dénes Á., Falshaw R., Itzhaki R. (2015). Anti-HSV1 activity of brown algal polysaccharides and possible relevance to the treatment of Alzheimer’s disease. Int. J. Biol. Macromol..

[13] Oliyaei N., Moosavi-Nasab M., Mazloomi S.M. (2022). Therapeutic activity of fucoidan and carrageenan as marine algal polysaccharides against viruses. 3 Biotech..

[14] Hayashi K., Lee J.B., Nakano T., Hayashi T. (2013). Anti-influenza A virus characteristics of a fucoidan from sporophyll of Undaria pinnatifida in mice with normal and compromised immunity. Microbes Infect..

[15] Liu H.D., Ma D.Y., Shi S.R., Song S.L., Li W.L., Qi X.H., Guo S.D. (2025). Preparation and bioactivities of low-molecular weight fucoidans and fuco-oligosaccharides: A review. Carbohydr. Polym..

[16] Sun X., Yan C., Fu Y., Ai C., Bi J., Lin W., Song S. (2024). Orally administrated fucoidan and its low-molecular-weight derivatives are absorbed differentially to alleviate coagulation and thrombosis. Int. J. Biol. Macromol..

[17] Sun Z., Wu Y., Yang B., Zhu B., Hu S., Lu Y., Zhao B., Du S. (2018). Inhibitory influence of Panax notoginseng saponins on Aspirin hydrolysis in human intestinal Caco-2 Cells. Molecules.

[18] Ahmad T., Eapen M.S., Ishaq M., Park A.Y., Karpiniec S.S., Stringer D.N., Sohal S.S., Fitton J.H., Guven N., Caruso V. (2021). Anti-Inflammatory Activity of Fucoidan Extracts In Vitro. Mar. Drugs.

[19] Cho M.L., Lee B.Y., You S.G. (2010). Relationship between oversulfation and conformation of low and high molecular weight fucoidans and evaluation of their in vitro anticancer activity. Molecules.

[20] Park S.B., Chun K.R., Kim J.K., Suk K., Jung Y.M., Lee W.H. (2010). The differential effect of high and low molecular weight fucoidans on the severity of collagen-induced arthritis in mice. Phytother. Res..

[21] Ulbricht C., Chao W., Costa D., Nguyen Y., Seamon E., Weissner W. (2009). An evidence-based systematic review of green-lipped mussel (Perna canaliculus) by the Natural Standard Research Collaboration. J. Diet. Suppl..

[22] Lawson B.R., Belkowski S.M., Whitesides J.F., Davis P., Lawson J.W. (2007). Immunomodulation of murine collagen-induced arthritis by N, N-dimethylglycine and a preparation of Perna canaliculus. BMC Complement. Altern. Med..

[23] Cardim Lessa R., Ebrahimi B., Jury J., Sewell M., Xie Y., Li Y., Lu J. (2024). Characterising bioactive components of green-lipped mussel via two extraction methods: In vitro assessment of antioxidant and immunomodulatory effects. Int. J. Food Sci. Technol..

[24] Mani S., Lawson J.W. (2006). In vitro modulation of inflammatory cytokine and IgG levels by extracts of Perna canaliculus. BMC Complement. Altern. Med..

[25] McPhee S., Hodges L.D., Wright P.F., Wynne P.M., Kalafatis N., Harney D.W., Macrides T.A. (2007). Anti-cyclooxygenase effects of lipid extracts from the New Zealand green-lipped mussel, Perna canaliculus. Comp. Biochem. Physiol. B Biochem. Mol. Biol..

[26] Whitehouse M.W., Macrides T.A., Kalafatis N., Betts W.H., Haynes D.R., Broadbent J. (1997). Anti-inflammatory activity of a lipid fraction (lyprinol) from the NZ green-lipped mussel. Inflammopharmacology.

[27] Darwesh A.M., Bassiouni W., Sosnowski D.K., Seubert J.M. (2021). Can N-3 polyunsaturated fatty acids be considered a potential adjuvant therapy for COVID-19-associated cardiovascular complications?. Pharmacol. Ther..

[28] De Cosmi V., Mazzocchi A., Turolo S., Syren M.L., Milani G.P., Agostoni C. (2022). Long-Chain Polyunsaturated Fatty Acids Supplementation and Respiratory Infections. Ann. Nutr. Metab..

[29] McGill A.R., Markoutsa E., Mayilsamy K., Green R., Sivakumar K., Mohapatra S., Mohapatra S.S. (2023). Acetate-encapsulated Linolenic Acid Liposomes Reduce SARS-CoV-2 and RSV Infection. Viruses.

[30] Das U.N. (2018). Arachidonic acid and other unsaturated fatty acids and some of their metabolites function as endogenous antimicrobial molecules: A review. J. Adv. Res..

[31] Das U.N. (2020). Can Bioactive Lipids Inactivate Coronavirus (COVID-19)?. Arch. Med. Res..

[32] Parolini C. (2019). Effects of fish n-3 PUFAs on intestinal microbiota and immune system. Mar. Drugs.

[33] Kohn A., Gitelman J., Inbar M. (1980). Interaction of polyunsaturated fatty acids with animal cells and enveloped viruses. Antimicrob. Agents Chemother..

[34] Kohn A., Gitelman J., Inbar M. (1980). Unsaturated free fatty acids inactivate animal enveloped viruses. Arch. Virol..

[35] Feng Y., Wassie T., Gan R., Wu X. (2023). Structural characteristics and immunomodulatory effects of sulfated polysaccharides derived from marine algae. Crit. Rev. Food Sci. Nutr..

[36] Huang S., Taylor C.G., Zahradka P. (2022). Long Chain N3-PUFA Decreases ACE2 Protein Levels and Prevents SARS-CoV-2 Cell Entry. Int. J. Mol. Sci..

[37] Goc A., Niedzwiecki A., Rath M. (2021). Polyunsaturated ω-3 fatty acids inhibit ACE2-controlled SARS-CoV-2 binding and cellular entry. Sci. Rep..

[38] Ebrahimi B., Baroutian S., Li J., Zhang B., Ying T., Lu J. (2023). Combination of marine bioactive compounds and extracts for the prevention and treatment of chronic diseases. Front. Nutr..

[39] Ebrahimi B., Lessa R.C., Baroutian S., Zhou Q., Chen X., Lu J. (2025). Antioxidant and immunomodulatory activity of Perna canaliculus oil extract in combination with low molecular weight fucoidan extracted from Undaria pinnatifida. Future Foods.

[40] Lessa R.C., Ebrahimi B., Li H., Guan X., Li Y., Lu J. (2024). Investigation of the In Vitro Immunomodulatory Effects of Extracts from Green-Lipped Mussels (Perna canaliculus). Nutraceuticals.

[41] Bi D., Yu B., Han Q., Lu J., White W.L., Lai Q., Cai N., Luo W., Gu L., Li S. (2018). Immune Activation of RAW264.7 Macrophages by Low Molecular Weight Fucoidan Extracted from New Zealand Undaria pinnatifida. J. Agric. Food Chem..

[42] Lu J., Shi K.K., Chen S., Wang J., Hassouna A., White L.N., Merien F., Xie M., Kong Q., Li J. (2018). Fucoidan Extracted from the New Zealand Undaria pinnatifida-Physicochemical Comparison against Five Other Fucoidans: Unique Low Molecular Weight Fraction Bioactivity in Breast Cancer Cell Lines. Mar. Drugs.

[43] Kowarz E., Löscher D., Marschalek R. (2015). Optimized Sleeping Beauty transposons rapidly generate stable transgenic cell lines. Biotechnol. J..

[44] Grosche L., Dohner K., Duthorn A., Hickford-Martinez A., Steinkasserer A., Sodeik B. (2019). Herpes Simplex Virus Type 1 Propagation, Titration and Single-step Growth Curves. Bio-Protocol.

[45] Hotard A.L., Shaikh F.Y., Lee S., Yan D., Teng M.N., Plemper R.K., Crowe J.E., Moore M.L. (2012). A stabilized respiratory syncytial virus reverse genetics system amenable to recombination-mediated mutagenesis. Virology.

[46] Hwang J.-H., Oh Y.-S., Lim S.-B. (2014). Anti-inflammatory activities of some brown marine algae in LPS-stimulated RAW 264.7 cells. Food Sci. Biotechnol..

[47] Crawford K.H.D., Eguia R., Dingens A.S., Loes A.N., Malone K.D., Wolf C.R., Chu H.Y., Tortorici M.A., Veesler D., Murphy M. (2020). Protocol and Reagents for Pseudotyping Lentiviral Particles with SARS-CoV-2 Spike Protein for Neutralization Assays. Viruses.

[48] Keyaerts E., Vijgen L., Maes P., Neyts J., Van Ranst M. (2004). In vitro inhibition of severe acute respiratory syndrome coronavirus by chloroquine. Biochem. Biophys. Res. Commun..

[49] Remali J., Aizat W.M. (2020). A Review on Plant Bioactive Compounds and Their Modes of Action Against Coronavirus Infection. Front. Pharmacol..

[50] Ma L., Yao L. (2020). Antiviral Effects of Plant-Derived Essential Oils and Their Components: An Updated Review. Molecules.

[51] Ho Y.J., Liu F.C., Yeh C.T., Yang C.M., Lin C.C., Lin T.Y., Hsieh P.S., Hu M.K., Gong Z., Lu J.W. (2018). Micafungin is a novel anti-viral agent of chikungunya virus through multiple mechanisms. Antivir. Res..

[52] Hans N., Malik A., Naik S. (2021). Antiviral activity of sulfated polysaccharides from marine algae and its application in combating COVID-19: Mini review. Bioresour. Technol. Rep..

[53] Kwon P.S., Oh H., Kwon S.J., Jin W., Zhang F., Fraser K., Hong J.J., Linhardt R.J., Dordick J.S. (2020). Sulfated polysaccharides effectively inhibit SARS-CoV-2 in vitro. Cell Discov..

[54] Song S., Peng H., Wang Q., Liu Z., Dong X., Wen C., Ai C., Zhang Y., Wang Z., Zhu B. (2020). Inhibitory activities of marine sulfated polysaccharides against SARS-CoV-2. Food Funct..

[55] Sun H., Xu J., Zhang G., Han J., Hao M., Chen Z., Fang T., Chi X., Yu C. (2022). Developing Pseudovirus-Based Neutralization Assay against Omicron-Included SARS-CoV-2 Variants. Viruses.

[56] Yim S.K., Kim K., Kim I.H., Chun S.H., Oh T.H., Kim J.U., Kim J.W., Jung W.H., Moon H.S., Ku B.S. (2021). Inhibition of SARS-CoV-2 Virus Entry by the Crude Polysaccharides of Seaweeds and Abalone Viscera In Vitro. Mar. Drugs.

[57] Malhotra R., Ward M., Bright H., Priest R., Foster M.R., Hurle M., Blair E., Bird M. (2003). Isolation and characterisation of potential respiratory syncytial virus receptor(s) on epithelial cells. Microbes Infect..

[58] Groothuis J.R., Simoes E., Levin M.J., Hall C.B., Long C.E., Rodriguez W.J., Arrobio J., Meissner H.C., Fulton D.R., Welliver R.C. (1993). Prophylactic administration of respiratory syncytial virus immune globulin to high-risk infants and young children. N. Engl. J. Med..

[59] Jin W., Zhang W., Mitra D., McCandless M.G., Sharma P., Tandon R., Zhang F., Linhardt R.J. (2020). The structure-activity relationship of the interactions of SARS-CoV-2 spike glycoproteins with glucuronomannan and sulfated galactofucan from Saccharina japonica. Int. J. Biol. Macromol..

[60] Wang L., Oh J.Y., Jayawardena T.U., Jeon Y.J., Ryu B. (2020). Anti-inflammatory and anti-melanogenesis activities of sulfated polysaccharides isolated from Hizikia fusiforme: Short communication. Int. J. Biol. Macromol..

[61] Zayed A., El-Aasr M., Ibrahim A.S., Ulber R. (2020). Fucoidan Characterization: Determination of Purity and Physicochemical and Chemical Properties. Mar. Drugs.

[62] Harden E.A., Falshaw R., Carnachan S.M., Kern E.R., Prichard M.N. (2009). Virucidal activity of polysaccharide extracts from four algal species against herpes simplex virus. Antivir. Res..

[63] Damonte E.B., Matulewicz M.C., Cerezo A.S. (2004). Sulfated seaweed polysaccharides as antiviral agents. Curr. Med. Chem..

[64] Zhu W., Ooi V.E., Chan P.K., Ang P.O. (2003). Isolation and characterization of a sulfated polysaccharide from the brown alga Sargassum patens and determination of its anti-herpes activity. Biochem. Cell Biol..

[65] Spear P.G., Shieh M.T., Herold B.C., WuDunn D., Koshy T.I. (1992). Heparan sulfate glycosaminoglycans as primary cell surface receptors for herpes simplex virus. Adv. Exp. Med. Biol..

[66] Sinha S., Astani A., Ghosh T., Schnitzler P., Ray B. (2010). Polysaccharides from Sargassum tenerrimum: Structural features, chemical modification and anti-viral activity. Phytochemistry.

[67] Chiang E.I., Syu J.N., Hung H.C., Rodriguez R.L., Wang W.J., Chiang E.R., Chiu S.C., Chao C.Y., Tang F.Y. (2022). N-3 polyunsaturated fatty acids block the trimethylamine-N-oxide-ACE2-TMPRSS2 cascade to inhibit the infection of human endothelial progenitor cells by SARS-CoV-2. J. Nutr. Biochem..

[68] Mounce B.C., Cesaro T., Carrau L., Vallet T., Vignuzzi M. (2017). Curcumin inhibits Zika and chikungunya virus infection by inhibiting cell binding. Antivir. Res..

[69] Wang X., Xia S., Zou P., Lu L. (2019). Erythromycin Estolate Inhibits Zika Virus Infection by Blocking Viral Entry as a Viral Inactivator. Viruses.

[70] Ebrahimi B., Lessa R.C., Lu S., Baroutian S., Suresh V., Zhou Q., Liu J., Lu J. (2025). Evaluation of the antidiabetic effects of green lipped mussel oil and its combination with low molecular weight fucoidan extracted from Undaria pinnatifida. Food Res. Int..

[71] Bao L., Deng W., Huang B., Gao H., Liu J., Ren L., Wei Q., Yu P., Xu Y., Qi F. (2020). The pathogenicity of SARS-CoV-2 in hACE2 transgenic mice. Nature.

[72] Bernstein D.I. (2020). Use of the Guinea pig model of genital herpes to evaluate vaccines and antivirals: Review. Antivir. Res..

[73] Song D., Lu C., Chang C., Ji J., Lin L., Liu Y., Li H., Chen L., Chen Z., Chen R. (2024). Natural Binary Herbal Small Molecules Self-Assembled Nanogel for Synergistic Inhibition of Respiratory Syncytial Virus. ACS Biomater. Sci. Eng..

